# Carbohydrate intakes, food sources and tracking in Australian young children

**DOI:** 10.1017/S0007114524002198

**Published:** 2024-10-28

**Authors:** Tinsae Shemelise Tesfaye, Ewa A. Szymlek-Gay, Karen J. Campbell, Miaobing Zheng

**Affiliations:** Institute for Physical Activity and Nutrition (IPAN), School of Exercise and Nutrition Sciences, Deakin University, Geelong, VIC, Australia

**Keywords:** Carbohydrate intake, Total sugar, Starch, Food sources, Tracking

## Abstract

Carbohydrate intake and key food sources of carbohydrates in early childhood are poorly understood. The present study described total carbohydrate intake and subtypes (i.e. starch, sugar), their primary food sources and their tracking among young Australian children. Data from children at ages 9 months (*n* 393), 18 months (*n* 284), 3·5 years (*n* 244) and 5 years (*n* 240) from the Melbourne InFANT Program were used. Three 24-hour recalls assessed dietary intakes. The 2007 AUSNUT Food Composition Database was used to calculate carbohydrates intake and food groups. Descriptive statistics summarised total carbohydrate and subtype intake and their main food sources. Tracking was examined using Pearson correlations of residualised scores between time points. Total carbohydrate, starch and sugar intakes (g/d) increased across early childhood. The percentage of energy from total carbohydrates (% E) remained stable overtime (48·4–50·5 %). From ages 9 months to 5 years, the %E from total sugar decreased from 29·4 % to 22·6 %, while the %E from starch increased from 16·7 % to 26·0 %. Sources of total carbohydrate intake changed from infant formula at 9 months to bread/cereals, fruits and milk/milk products at 18 months, 3·5 and 5 years. Across all time points, the primary sources of total sugar intake were fruit, milk/milk products and cakes/cookies, whereas main food groups for starch intake included bread/cereals, cakes/cookies and pasta. Weak to moderate tracking of total carbohydrates, total sugar and starch (g/d) was observed. These findings may have the potential to inform the refinement of carbohydrate intake recommendations and design of interventions to improve children’s carbohydrate intake.

The primary role of dietary carbohydrates is to provide energy to the body’s cells, organs and tissues, particularly the brain, which requires glucose for its metabolism^(1,2)^. They also play a crucial role in the growth and development of children^(3)^. Nevertheless, the consumption of simple carbohydrates with a high sugar content has been linked to poor overall dietary quality, an increased risk of obesity, chronic diseases and a higher risk of dental caries in children^(2,4)^. Conversely, consuming foods containing starch or complex carbohydrates such as whole grains, fruits, vegetables and pulses, along with high intakes of dietary fibre, has been shown to have a positive health impact^(2,4)^.

Carbohydrates can be classified in many ways and may be expressed using varying terminology. Nutritionally, carbohydrates can be categorised into two main groups: ‘digestible/glycaemic/available carbohydrates’ defined as those digested and absorbed in the human small intestine, providing carbohydrates to body cells, and ‘non-digestible/unavailable carbohydrates’ such as dietary fibre, defined as those passing to the large intestine, where they nourish intestinal bacteria^(3–5)^. Digestible carbohydrates are further classified into simple (sugar) and complex carbohydrates (starch)^(3,6)^. The term ‘sugars’ refers to both monosaccharides (galactose, fructose and glucose) and disaccharides (lactose and sucrose), which contribute to the sweet taste of our food^(4,6–8)^. In the literature, other classifications include sugars naturally occurring in foods (e.g. ‘intrinsic’ sugars) *v*. sugars added to foods during processing or preparation (e.g. ‘added’ or ‘extrinsic’ sugars’)^(4,8)^.

Even though carbohydrates are an essential energy source, contributing approximately half of the dietary energy intake in most countries worldwide, the intake of carbohydrates in children is poorly understood^(5)^. Studies investigating carbohydrate intake trends and key sources during early childhood are sparse and have been conducted in the USA^(9)^, Asia^(10)^ and European countries^(6,11)^. Previous studies on carbohydrate intake and main food sources in Australian children have primarily focused on total carbohydrates^(12,13)^, or specific types such as sugar^(14)^ or fibre^(15)^, with most utilising cross-sectional data only. Australia’s most recent national nutrition survey (2011–2012)^(16)^ revealed total carbohydrate, total sugar and starch intakes, as well as key food sources in children aged 2–8 years. However, it is important to note that the survey did not include children under 2 years of age.

Furthermore, evidence indicates dietary habits may be shaped at a young age, maintained throughout life with tracking over time and have enduring impact on health^(17,18)^. However, carbohydrate intake trends and tracking in early childhood remain unclear. Given the crucial role of carbohydrates in human health, there is an urgent need for longitudinal data on carbohydrate intakes, food sources and tracking to gain a deeper understanding of children’s carbohydrate intake in order to identify evidence-based solutions. Investigating trends in carbohydrate intakes, including subtypes like sugar and starch, and identifying main food sources can guide refinement of recommended intakes and food-based dietary guidelines for young Australian children, as well as inform the design of nutrition interventions to optimise carbohydrate intakes. Thus, the present study aimed to (1) describe the intakes of total carbohydrates and their subtypes (starch and total sugar), (2) describe their main food sources and (3) analyse tracking among young Australian children across the first 5 years of life (at 9 and 18 months and 3·5 and 5 years).

## Methods

### Study population

The present study is a secondary analysis of data from the Melbourne Infant Feeding Activity and Nutrition Trial (InFANT) Program^(19)^. Detailed study protocol and intervention outcomes were reported previously^(19–21)^. Briefly, it was a 15-month intervention study aimed at preventing childhood obesity among 542 first-time mothers and their children from when their infants were aged 4–18 months^(19)^. Without a specific focus on carbohydrates, nutrition education was provided to parents in the intervention group, while parents in the control group received standard care^(21)^. The cohort was then followed up with no intervention when the children were 3·5 and 5 years old^(20)^. Ethics approval was obtained from Deakin University Human Research Ethics Committee (ID no. EC 175-2007) and the Victorian Office for Children (ref: CDF/07/ 1138).

### Demographic and socio-economic measures

At study baseline (when the children were four months old), parents reported information on child sex, age, gestational age, maternal country of birth (Australia or other), maternal education, maternal employment status, maternal height and pre-pregnancy body weight^(22)^. Mothers reported a child’s birth weight based on birth records. Weight for gestational age was classified as small for gestational age if the child’s birth weight was below the 10th percentile, appropriate for gestational age if birth weight was between the 10th and 90th percentiles and large for gestational age if birth weight was above the 90th percentile^(23)^. At ages 4 and 18 months, mothers reported the timing of breast-feeding cessation and introduction of complementary foods^(19)^. Breast-feeding duration and timing of solid food introduction were then coded as dichotomous variables, using the cut-off of 6 months (< 6 months, ≥ 6 months), consistent with previous publications from this cohort^(15,24)^. Maternal education attainment was classified as below university (high school/certificate/diploma/trade) or university (university degree or higher). Self-reported maternal pre-pregnancy BMI was calculated as weight in kilograms divided by height in meters squared^(25,26)^.

### Child anthropometric characteristics

Trained research staff measured children’s length/height and weight at ages 9 and 18 months and 3·5 and 5·0 years^(21,26)^. Height/length was measured barefoot twice to the nearest 1 mm with (Seca 210; Seca Deutschland) or a portable stadiometer (Invicta IPO955; Oadby). Children’s weight was measured to the nearest 10 g with calibrated digital scales (Tanita 1582; Tanita Corp), wearing light clothes and without shoes. The mean of two measurements was used in the current analyses.

### Dietary assessment

Child’s dietary intake was assessed by telephone-administered multiple-pass three 24-hour recalls at ages 9 months, 18 months, 3·5 years and 5 years by trained nutritionists^(20,21)^. Parents of children were interviewed on 3 nonconsecutive days, including 2 weekdays and 1 weekend day. Ninety-six percent of the telephone calls were unscheduled to minimise response bias. A food measurement booklet consisting of photographs of standard portion sizes and examples of measurements were provided to parents to assist with portion size estimation^(25)^. Consistent with previous studies^(22,25)^, breast-feeding was recorded as minutes spent breast-feeding and then converted to volume using a conversion factor of 10 ml per minute up to a maximum of 100 ml for any one feed. If breast milk was expressed, the volumes estimated from caregiver reports were used^(22)^. The dietary data collected were converted into daily energy intake (kJ/d), total carbohydrate intake (g/d), total sugar intake (g/d) and starch intake (g/d) using the 2007 Australian Food, Supplement, and Nutrient Food Composition Database (AUSNUT 2007)^(27)^. The percentages of total carbohydrates, total sugar and starch in relation to total energy intake (% of energy) were also calculated. Food sources of total carbohydrates, total sugar and starch in the diet were categorised into thirty food groups (online Supplementary Table S1) following the standard food grouping in the 2007 AUSNUT database^(27)^. Mean values and standard deviations of daily intake of total carbohydrates (g/d), total sugar and starch, along with the respective percentage contributions to total energy intake, were computed for each food group. Aligned with previous approaches used in the Melbourne InFANT program^(22,25)^, this analysis used the mean daily dietary intake over 3 days.

### Statistical analysis

The current analyses included the data of children at ages 9 months,18 months, 3·5 years and 5 years (*n* 542). Data were excluded for children from non-first-time mothers (*n* 14) and those with fewer than 3 days of dietary recalls or outlier total energy intake (±3 standard deviations). Additional exclusions were made at 9 and 18 months in line with previous publication from the InFANT study^(22)^. Specifically, children younger than 7 months or older than 11 months were excluded from the analysis at age 9 months (*n* 30), while those younger than 16 months or older than 20 months were excluded from the sample at age 18 months (*n* 85). No age exclusion was applied to 3·5 and 5 year follow-ups. The age ranges at each time point for the current analysis are 7–10 months, 15–29 months, 3–4 years and 4–5 years. After implementing these exclusion criteria, the final analysis included samples of *n* 393 at 9 months, *n* 284 at 18 months, *n* 244 at 3·5 years and *n* 240 at 5 years (online Supplementary Fig. S1). Since there were no statistical differences observed in total carbohydrate, sugar and starch intakes between the intervention and control groups apart from sugar intakes at age 18 months (online Supplementary Table S2), the data from both groups were pooled for the current analyses. The present study used *t* tests or ANOVA to compare total carbohydrate, total sugar and starch intake by cohort characteristics. Descriptive statistics were used to summarise daily intakes of energy (kJ/d), total carbohydrates (g/d), carbohydrate intake per body weight (g/kg/d), % of energy from carbohydrates and key food sources of carbohydrate intake at all time points. Similarly, intakes of total sugar and starch (g/d), % of energy from total sugar and starch and key food sources at all time points were calculated. Frequencies and proportions were reported for categorical variables, while means and standard deviations (sd) were presented for continuous variables.

To assess tracking across all time points, residualised intake scores were created by regressing the children’s carbohydrate and subtypes (total sugar and starch) intake after adjusting for age, sex and total energy intake^(28)^. Pearson product–moment correlations were calculated to the adjusted residual carbohydrate intake scores between different time points. Statistical assumptions were satisfied for all the models examined. Further, sensitivity analyses were conducted to evaluate tracking by including only the samples followed at all time points (9 months, 18 months, 3·5 years and 5 years). Consistent with previous publications^(22,25)^, the interpretation of these correlation coefficients was based on the following recommendations: < 0·3 for weak tracking, 0·3–0·6 for moderate tracking and > 0·6 for high tracking^(29)^. Stata v17 (StataCorp LLC) was used for all the statistical analyses.

## Results

Of 393 children, 52·4 % of participants were boys, and the majority of the children had an appropriate birth weight for gestational age (82·7 %). Most of the children were breastfed for more than 6 months (75·9 %) and introduced to solid foods at or after the age of 6 months (90·9 %). Most mothers (79·3 %) were born in Australia, 7·2 % were in paid employment and 58·2 % had a university education.

Comparisons of total carbohydrate, total sugar and starch intakes (g/d) at age 9 months by child and maternal characteristics are shown in Table 1. There was a statistically significant difference in mean carbohydrate, total sugar and starch intakes between boys and girls, with higher intakes observed in boys than in girls (*P* ≤ 0·001). No significant difference was observed for total carbohydrates, total sugar and starch by child birthweight. Compared with children who were breastfed for 6 months or more and introduced to solids after 6 months of age, those who were breastfed for < 6 months and introduced to solids early (< 6 months) had higher total carbohydrate (*P* ≤ 0·001), total sugar (*P* ≤ 0·001) and starch intakes (*P* ≤ 0·001). The mean total sugar intakes of children whose mothers were in paid employment were significantly higher than those of children whose mothers were unemployed (*P* = 0·02). Similarly, children with mothers below a university degree had higher total carbohydrate and starch intakes than children whose mothers had a university degree or higher (*P* < 0·001). However, no significant difference was observed in the total sugar intake of children by maternal education status.

**Table 1.** Comparison of mean (sd) total carbohydrate, total sugar and starch (g/d) intake at age 9 months by child and maternal characteristics (*n* 393) at 9 months in the Melbourne infant feeding activity and nutrition trial program (Mean values and standard deviations)

Variables	n*	%	Total CHO intakes		*P* value^†^	Total sugar intakes		*P* value	Starch intakes		*P* value
			Mean	sd		Mean	sd		Mean	sd	
Intervention group											
Control	194	49·4	99·8	25·2	0·89	64·0	14·5	0·36	34·8	16·1	0·61
Intervention	199	40·4	99·4	26·2		62·7	14·8		35·7	16·2	
Child Sex											
Boy	206	52·4	106·0	26·2	< 0·001	66·7	14·9	< 0·001	38·2	17·3	< 0·001
Girl	187	47·6	92·7	23·3		59·7	13·4		32·0	14·2	
Birthweight for gestational age											
SGA	33	8·4	99·5	27·5	0·96	64·1	15·5	0·47	34·6	15·8	0·53
AGA	325	82·7	99·7	25·9		63·0	14·5		35·5	16·6	
LGA	35	8·9	99·5	22·4		65·6	15·5		33·0	12·1	
Breast-feeding duration											
< 6 months	91	24·1	110·2	20·7	< 0·001	68·4	13·1	< 0·001	40·9	14·1	< 0·001
≥ 6 months	287	75·9	96·1	26·5		61·6	14·9		33·4	16·5	
Timing of solid food introduction											
< 6 months	35	9·02	117·8	4·3	< 0·001	70·1	14·4	0·004	46·4	20·9	< 0·001
≥ 6 months	353	90·9	97·7	25·0		62·5	14·5		34·1	15·7	
Maternal country of birth											
Australia	311	79·3	100·1	25·4	0·59	64·1	14·9	0·06	34·9	15·3	0·37
Other	81	20·7	98·3	27·2		60·7	13·3		36·8	19·2	
Maternal current working status^‡^											
Unemployed	361	92·8	99·3	25·6	0·23	63·0	14·4	0·02	35·3	16·3	0·88
Employed	28	7·2	105·4	26·8		69·5	16·2		35·0	16·2	
Maternal education											
Below University	164	41·8	104·0	26·7	< 0·001	64·5	14·7	0·19	38·5	17·7	< 0·001
University degree or higher	228	58·2	96·5	24·6		62·5	14·7		32·9	14·6	

CHO, carbohydrate. SGA, small for gestational age, AGA, appropriate for gestational age, LGA, large for gestational age.

* n differs for each variable due to missing data.

† Differences between CHO, total sugar and starch were assessed by *t* test or ANOVA,

‡ employed: (part time /full time paid employment at time of data collection), unemployed (student/maternity leave/home duties/unemployed).

### Dietary carbohydrate intake

Dietary energy, total carbohydrate, starch and total sugar intakes over four time points are described in Table 2. From ages 9 months to 5 years, the mean daily energy and total carbohydrate intake (g/d) increased from 3490 kJ/d to 5889 kJ/d and 99·7–174 g/d, respectively. Likewise, total sugar and starch intakes (g/d) increased throughout early childhood. Total carbohydrate intake per kg body weight appeared to be steady from 9 to 18 months of age but decreased afterward. The proportion of total carbohydrates to total energy remained stable overtime (48·4–50·5 %). Nevertheless, the percentage of energy from total sugar decreased from 29·4 % at 9 months to 22·6 % at 5 years, while the percentage of energy from starch increased from 16·7 % at 9 months to 26·0 % at 5 years.

**Table 2.** Energy, total carbohydrate and starch intake at 9 months, 18 months, 3·5 years and 5 years in Melbourne infant feeding activity and nutrition trial program^*^ (Mean values and standard deviations)

	9 months (*n* 393)		18 months (*n* 284)		3·5 years (*n* 244)		5 years (*n* 240)	
	Mean	sd	Mean	sd	Mean	sd	Mean	sd
Energy (kJ/d)	3490	822	4411	832	5314	1090	5889	1213
Total carbohydrate (g/d)	99·7	25·7	128·3	27·0	156·7	35·3	174·0	37·2
Total sugar(g/d)	63·4	14·7	68·6	17·6	78·5	22·8	83·0	25·7
Starch (g/d)	35·2	16·2	58·6	19·5	76·6	21·9	89·1	23·3
Carbohydrate per body weight (g/kg/d)	11·3	2·9	11·5	2·6	9·5	2·3	8·7	2·1
Total energy (%)								
Total carbohydrate	48·4	4·7	49·6	5·4	50·3	6·3	50·5	5·8
Total sugar	29·4	4·2	25·0	4·9	23·7	5·2	22·6	5·2
Starch	16·7	5·3	22·6	6·2	24·6	5·5	26·0	5·4

* Nutrient reference values (NRV) for carbohydrates (adequate intake (AI): age 0–6 months: 60 g/d, age 7–12 months: 95 g/d), no NRV for age 1–5 years, acceptable macronutrient distribution range (AMDR) for carbohydrates is 45–65 % of energy for all time points.

### Food sources of total carbohydrate, total sugar and starch

At the age of 9 months, the predominant source of total carbohydrates (24 %) in infants was infant formula, followed by breads/cereals (16·1 %), fruits (12·9 %), breast milk (10·5 %) and milk and milk products (6·9 %) (Table 3). At 18 months, 3·5 years and 5 years, the major sources of total carbohydrates shifted to breads/cereals, followed by fruits, milk and milk products and cakes/cookies. Other key contributors to total carbohydrate intake, ranging from 4 % to 5 %, included infant cereals, infant foods, pasta, sweet snacks and sugar-sweet beverages.

**Table 3.** Main total carbohydrate food sources at ages 9 months, 18 months, 3·5 years and 5 years in Melbourne infant feeding activity and nutrition trial program (Percentages; mean values and standard deviations)

		9 months (*n* 393)							18 months (*n* 284)							3·5 years (*n* 244)							5 years (*n* 240)					
	% consumers*	% of total energy		% of total CHO		CHO provided (g)		% consumers*	% of total energy		% of total CHO		CHO provided (g)		% consumers*	% of total energy		% of total CHO		CHO provided (g)		% consumers*	% of total energy		% of total CHO		CHO provided (g)	
Food Group		Mean	sd	Mean	sd	Mean	sd		Mean	sd	Mean	sd	Mean	sd		Mean	sd	Mean	sd	Mean	sd		Mean	sd	Mean	sd	Mean	sd
Breast milk	45	6·4	8·5	10·5	14·9	11	13·9	10	0·6	2·5	0·8	37	1·6	6·1	1	0·0	0·0	0·0	0·0	0·0	0·1	0	0		0	0	0	0
Infant/toddler formula	70	15·0	12·1	24·0	20·5	32	26·4	13	1·2	3·8	1·5	49	3·0	9·3	1	0·1	1·4	0·2	1·9	0·4	4·2	1	0·1	1·3	0·1	1·7	0·4	5·4
Milk and milk products^†^	91	4·6	3·9	6·9	5·9	9·5	8·5	98	12·9	6·2	17·4	9·2	33	16·8	98	10·9	5·3	13·2	6·6	34	18·2	98	9·6	4·9	11·1	5·9	33	18·2
Breads/cereals	94	11·1	8·8	16·1	10·8	23	21·8	100	21·9	10·4	28	11·5	57	28·3	100	25	10·7	29	11·0	78	35·7	100	26·8	10·9	30	10·8	92	40·8
Pasta	44	2·2	3·6	3·2	4·9	4·7	8·3	58	3·8	4·9	4·8	5·9	10	13·2	57	4·5	5·7	5·2	6·5	14·3	18·9	55	4·2	5·4	4·6	5·5	14	18·1
Infant cereals	76	3·4	3·9	5·3	5·8	6·9	8·5	22	0·5	1·3	0·7	1·7	1·3	3·1	1	0·0	0·3	0·0	0·4	0·1	1·0	0	0	0	0	0	0	0
Infant food	64	4·1	4·4	6·2	6·6	8·5	9·2	36	1·6	2·8	2·1	3·6	4·2	7·2	9	0·4	1·2	0·5	1·6	1·2	4·1	3	0·1	0·7	0·1	0·9	0·3	2·3
Cakes/cookies	48	1·7	3·4	2·5	4·7	3·7	8·3	88	7·3	6·9	9·2	8·1	19	18·8	95	10·9	7·7	12·7	8·5	34	24·6	96	11·9	7·2	13·6	7·9	42	26·9
Savoury snacks	2	0·0	0·1	0·0	0·1	0·0	0·2	15	3·3	1·2	0·4	1·5	0·8	3·1	76	0·8	2·0	1·0	2·4	2·6	6·2	49	1·6	2·3	1·8	2·7	5·5	8·4
Sweet snacks	6	0·2	0·9	0·3	1·2	0·5	2·3	35	2·3	4·7	2·8	5·5	5·8	11·7	76	4·5	4·9	5·2	5·3	14	15·3	74	4·8	5·7	5·3	5·7	16	19·1
Butter/oil/fat spreads	35	0·0	0·0	0·0	0·1	0·0	0·1	78	0·0	0·1	0·0	0·1	0·1	0·3	79	0·0	0·1	0·0	0·1	0·1	0·3	85	0·0	0·3	0·1	0·3	6·5	6·5
Condiments	45	0·2	0·6	0·4	0·9	0·5	1·4	73	0·6	0·9	0·7	1·2	1·5	2·4	80	0·9	1·2	0·9	1·2	2·6	3·8	85	0·8	0·9	0·9	1·2	2·8	3·3
Sugar based accompaniments	8	0·1	0·7	0·2	0·9	0·3	1·5	80	0·8	1·6	0·9	2·0	1·9	4·3	51	1·6	2·8	1·8	3·2	4·9	8·8	58	2·1	4·8	2·2	4·1	6·9	13·3
Sugar-sweetened beverage	7	0·1	0·6	0·1	0·8	0·2	0·9	23	0·8	2·1	1·1	2·6	1·9	4·8	60	3·2	4·7	3·6	5·2	9·7	14·6	67	3·1	4·0	3·4	4·3	11	13·8
Beef, veal, lamb	35	0·5	0·9	0·7	1·6	0·9	2·1	49	0·6	1·2	0·8	1·6	1·7	3·5	40	0·5	1·1	0·6	1·4	1·6	3·4	39	0·6	1·2	0·7	1·5	2·0	4·5
Poultry	24	0·5	1·6	0·7	2·0	1·0	3·9	37	0·6	1·4	0·8	1·8	1·5	3·5	41	0·8	1·7	0·9	1·9	2·5	5·8	43	1·2	2·1	1·3	2·4	3·9	7·4
Egg and egg dishes	10	0·1	0·6	0·1	0·9	0·2	1·6	28	0·3	1·4	0·4	1·6	0·9	4·0	21	0·2	1·2	2·2	1·4	0·6	3·6	38	0·3	1·2	0·3	1·5	0·9	4·2
Fish	10	0·12	0·7	0·2	1·1	0·3	1·5	22	0·5	1·5	0·7	1·8	1·4	3·8	52	0·5	1·4	0·7	1·8	1·7	4·6	24	0·6	1·5	0·7	1·7	2·3	5·3
Potato	42	1·3	2·2	1·8	3·0	0·3	1·5	47	1·7	2·9	2·2	3·8	4·5	7·9	67	2·9	3·4	3·4	3·9	9·2	11·1	67	2·9	3·6	3·3	3·9	9·8	11·1
Legumes	17	0·5	1·5	0·6	2·0	0·9	3·3	26	0·9	2·4	1·2	3·0	2·5	6·4	22	0·6	1·5	0·6	1·7	1·8	4·7	22	0·7	1·9	0·7	1·9	2·3	6·2
Soup	17	0·5	1·5	0·7	2·1	1·1	3·4	22	0·8	1·9	1·0	2·4	2·0	4·9	16	0·6	2·2	0·7	2·3	2·1	8·7	20	0·8	1·9	0·9	2·1	2·6	6·5
Fruit	95	8·6	6·5	12·9	9·3	17	13·7	99	14·0	7·0	17·9	8·2	36	19·1	99	13·1	6·7	15·3	7·2	40	21·8	99	12·8	6·3	14·4	6·7	43	21·4
Vegetables	94	3·1	2·9	6·1	5·4	8·4	8·8	90	1·4	1·9	3·5	4·6	6·9	10·9	93	0·7	0·8	2·2	2·5	6·0	7·5	95	0·8	0·9	2·6	2·6	7·8	7·8

CHO: Total carbohydrates;

* Percentage of children who consumed food at least once during data collection.

† Includes dairy milk, yoghurt, cheese, frozen milk products and custard.

**Fig. 1.**
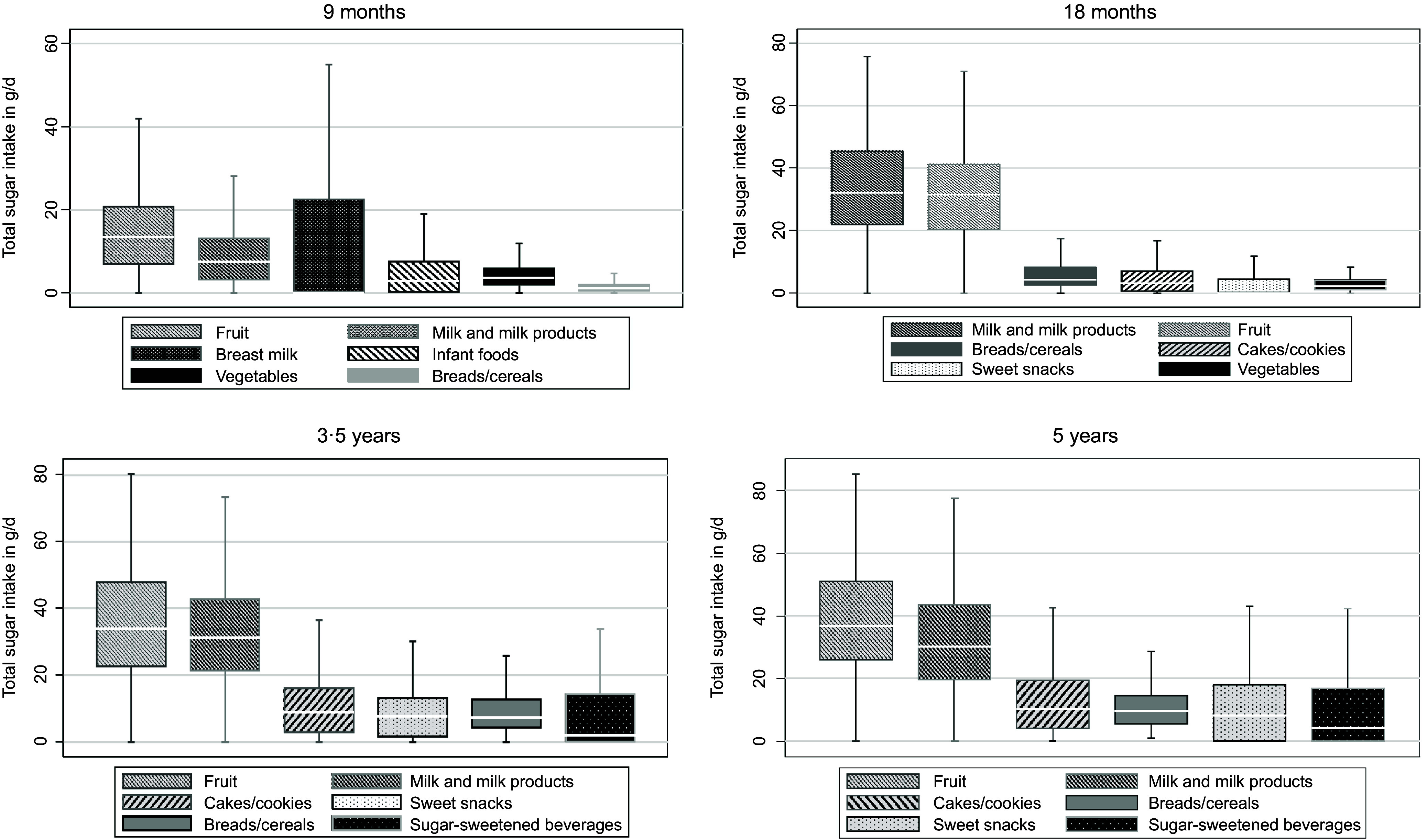
Main total sugar food sources at ages 9 months (*n* 393), 18 months (*n* 284), 3·5 years (*n* 244) and 5 years (*n* 240) in Melbourne Infant Feeding Activity and Nutrition Trial Program.

**Fig. 2.**
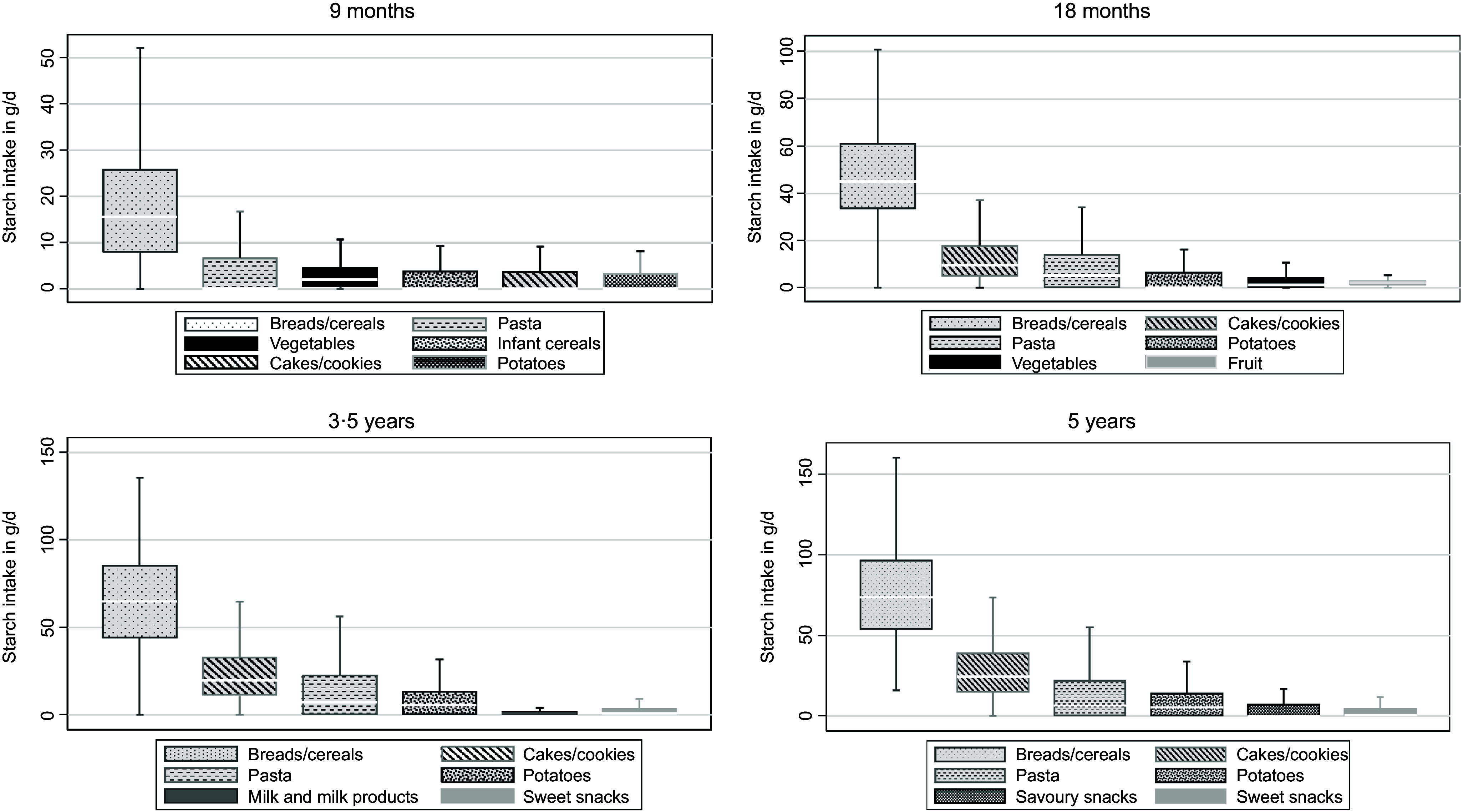
Main starch food sources at ages 9 months (*n* 393), 18 months (*n* 284), 3·5 years (*n* 244) and 5 years (*n* 240) in Melbourne Infant Feeding Activity and Nutrition Trial Program.

Regarding total sugar intake, fruits were the primary source of total sugar at all time points (28·5–33·1 %), followed by milk and milk products (18·4–33·4 %) (Fig. 1). Breads/cereals, cakes/cookies, sugar-sweetened beverages and sweet snacks were important food groups each contributing approximately 3–4 % of total sugar intake. Finally, across all time points the primary sources of starch intake were breads/cereals (26·4–52·5 %) followed by cakes/cookies (6·8–17·7 %), pasta (8·4–9·9 %) and potatoes (4·5–6·2 %) (Fig. 2).

### Tracking of total carbohydrates, sugar and starch intakes

Residualised total carbohydrate intake at 9 and 18 months showed a weak yet statistically significant correlation (*r* = 0·24; *P* ≤ 0·001). In contrast, no tracking was observed for carbohydrate intake between 9 months and 3·5 years (*r* = 0·07; *P* = 0·29), or between 9 months and 5 years (*r* = 0·06; *P* = 0·37) (Table 4). Similar weak correlations at ages 9 and 18 months were observed for total sugar (*r* = 0·15; *P* = 0·02) and starch intake (*r* = 0·18; *P* = 0·004). No evidence of tracking was found for sugar or starch between 9 months and 3·5 years (*r* = 0·11; *P* = 0·12; *r* = 0·12; *P* = 0·08), or for starch between 9 months and 5 years (*r* = 0·09; *P* = 0·15). Additionally, residualised total carbohydrate intake at 18 months showed a moderate correlation with scores at 3·5 years (*r* = 0·30; *P* ≤ 0·001) and a weak correlation at 5 years (*r* = 0·15; *P* = 0·05). However, residualised total sugar and starch intakes at 18 months significantly correlated with intakes at 3·5 and 5 years of age (*r* ranging from 0·23–0·34). A moderate and highly significant correlation was observed at ages 3·5 years and 5 years for total carbohydrate (*r* = 0·39; *P* < 0·0001), total sugar (*r* = 0·39; *P* ≤ 0·0001) and starch intakes (*r* = 0·41; *P* ≤ 0·001).

**Table 4.** Tracking of total carbohydrate, sugar and starch at ages 9 months, 18 months, 3·5 years and 5 years in Melbourne infant feeding activity and nutrition trial program

	9 months			18 months			3·5 years			5 years		
	Tracking (*P*-value)^*^			Tracking (*P*-value)			Tracking (*P*-value)			Tracking (*P*-value)		
	CHO	Sugar	Starch	CHO	Sugar	Starch	CHO	Sugar	Starch	CHO	Sugar	Starch
18 months												
CHO	0·24 (*P* < 0·001)											
Sugar		0·15 (0·02)										
Starch			0·18·(0·004)									
3·5 years												
CHO	0·07 (0·29)			0·29 (*P* < 0·001)								
Sugar		0·11 (0·12)			0·29 (*P* < 0·001)							
Starch			0·12 (0·08)			0·34 (*P* < 0·001)						
5 years												
CHO	0·06 (0·37)			0·15 (0·05)			0·39 (*P* < 0·001)					
Sugar		0·15 (0·03)			0·23 (0·002)			0·39 (*P* < 0·001)				
Starch			0·09 (0·15)			0·18 (0·02)			0·41 (*P* < 0·001)			

CHO, total carbohydrates.

* Pearson correlation of linear regression predicted residuals of carbohydrates, total sugar and starch at each timeline, *n* at each compared age is as follows: 9 months and 18 months (*n* 256), 9 months and 3·5 years (*n* 213), 9 and 5 years (*n* 211), 18 months and 3·5 years (*n* 178), 18 months and 5 years (*n* 175) and 3·5 and 5 years (*n* 204).

### Sensitivity analysis

When evaluating carbohydrate, sugar and starch intake tracking using samples followed at all time points (9 months, 18 months, 3·5 years and 5 years) (*n* 141), broadly similar correlations were observed, with some exceptions (see online Supplementary Table S6). Weak correlation was found for carbohydrate intake between 9 months and 3·5 years (*r* = 0·21; *P* = 0·01), while no significant correlations were observed for sugar (*r* = 0·10; *P* = 0·23) and starch (*r* = 0·12; *P* = 0·15) intake between 9 months and 18 months.

## Discussion

In a cohort of Australian young children, the intakes of total carbohydrates and subtypes (sugar, starch) (g/d) increased over time in the first years of life. The proportion of total carbohydrates to total energy intake remained relatively stable across all time points. However, the percentage of energy from total sugar decreased, while the percentage of energy from starch increased. Sources of total carbohydrates intake changed over time, shifting from infant formula to breads/cereals, fruits, milk and milk products and cakes/cookies. Regarding total sugar intake, fruits and milk and milk products were the key food sources over time. However, discretionary foods, such as cakes/cookies and sweet snacks, gained importance at ages 3·5 and 5 years. Food groups that contributed most to starch intake were breads/cereals, cakes/cookies and pasta. Weak to moderate tracking of total carbohydrate, total sugar and starch was observed across the first 5 years of life.

Our study is the first to use longitudinal data to report total carbohydrate, total sugar and starch intakes from ages 9 months to 5 years. The total carbohydrate intake (100–170 g/d) observed in our cohort is similar to those (111–192 g/d) reported in a cross-sectional study of USA children from age 1 to 5 years^(30)^. Similarly, findings from a longitudinal cohort conducted in European countries (Belgium, Germany, Italy, Poland and Spain) demonstrated an increase in total carbohydrate intake with age, from 150 g/d at 3 years to 192 g/d at 8 years^(31)^. In the current study, the proportion of total energy intake from dietary carbohydrates ranged from 48·4 to 50·5 %. Previous studies conducted in Germany^(32)^ and other European countries (Belgium, Italy, Poland and Spain)^(31)^ showed that the percentage contribution of total carbohydrates to energy ranged from 51·0 to 53·2 % among young children. It is crucial to note that this European study^(31)^ involved older children aged 3–8 years and used weighed food records to collect dietary intakes. Therefore, our findings are not directly comparable with these studies due to differences in age, dietary assessment methods and country-specific food composition tables.

Consistent with two reviews that examined sugar^(3,33)^ and starch consumption^(3)^ in young children across different countries, our study revealed that the percentage of energy from total sugars is high in infancy (29·4 %) but gradually decreased throughout early childhood, reaching 22·6 % at age 5. Meanwhile, the percentage of energy from starch increased from 16·7 % at 9 months to 26·0 % at 5 years. The observed trend can be explained by the high consumption of breast milk in infancy, which was the major contributor to total sugar intake, following fruits and milk/milk products. For example, in our study, at 9 months of age, breast milk ranked as the third largest contributor to total sugar, contributing 18 % of total sugar. Unsurprisingly, by 18 months, the percentage of total sugar from breast milk had declined, contributing 1·6 % to the overall total sugar intake. This change might be attributed to the progressive replacement of breast milk with complementary foods, resulting in children obtaining their energy from a wider variety of sources.

Many national and regional authorities, such as the WHO^(34)^, the European Food Safety Authority^(4)^ and guidelines from the United Kingdom (UK)^(35)^, the USA, Canada^(36)^ and Australia^(1)^, have established dietary recommendations for carbohydrate intake for children in the form of the recommended proportion of total energy from carbohydrates. However, the specific figures/ ranges differ amongst these authorities, ranging from a lower limit of 40 % energy to 55–75 % energy. Dietary guidelines in the USA^(36)^ also provide Adequate Intake and Recommended Dietary Allowance for children aged 0–12 months and 1–8 years, respectively. Similarly, adequate intake for children aged 0–12 months is provided in Australia^(1)^. However, these values are subject to ambiguity and ongoing debate as they have been determined from the nutrient composition of breast milk obtained from a small sample of USA lactating women and rely on information primarily tailored for adults^(1,36)^. The review of international recommendations on carbohydrate intakes by Buyken *et al.* ^(37)^ also showed a wide variation in the methods used to derive these guidelines, including differences in terminology and carbohydrates classifications, as well as variations in the choice and number of selected health outcomes. Similarly, there is inconsistency among quantitative dietary recommendations for sugar intakes across different authorities^(37)^. This variation and lack of uniformity within the expert community adversely impact the acceptance and implementation of recommendations. Additionally, this poses a challenge in evaluating children’s adherence to dietary carbohydrate intake guidelines.

The current study is one of the few studies to examine key food sources of total carbohydrates, starch and total sugar intake during early childhood. Consistent with findings from Belgium^(11)^ and Spain^(6)^, main sources of total carbohydrates in young children were breads/cereals, milk and milk products and cakes/cookies. In addition, our study showed that the predominant sources of total sugar intake were fruits, milk and milk products, broadly consistent with previous studies^(6)^. Naturally occurring sugars from fruits and milk products, which contributed to the highest percentage to the total sugar intake in the present study, are less concerning, as there is no sound evidence of adverse effects from an excessive intake of intrinsic sugars (naturally occurring sugars)^(38,39)^. Indeed, a review by European Society for Pediatric Gastroenterology, Hepatology and Nutrition Committee suggested sugars should preferably be consumed in its natural form such as human milk, milk, unsweetened dairy products and fresh fruits^(40)^. In addition, intrinsic sugars are more likely to be present in foods alongside useful nutrients such as fibre, vitamins and minerals^(40)^. In contrast, a study conducted among USA infants and toddlers aged 0–24 months^(9)^ revealed a higher percentage of total sugar originating from juices, sugar-sweetened beverages and sweet bakery products, higher than the contributions outlined in the present study. Despite these food groups having a lower contribution in our study, their intake increased at later ages (3·5 and 5 years). The increased consumption of sugar from discretionary foods as children age might suggest that children gain more autonomy over their dietary choices, while parents may find it more challenging to promote healthier diet options^(41)^.

Breads/cereals contributed to the majority of starch intakes at ages 9 and 18 months and 3·5 and 5 years in our study (45–53 %). High starch contributions from cereals also have previously been observed in Spanish children^(6)^. The role of starch from breads/cereals in health varies by the specific types of breads/cereals consumed. Wholegrain breads/cereals contain more fibre, vitamins, minerals and antioxidants than refined cereal foods such as white bread because many of the nutrients occur in the outer layer of the grain, which is lost during processing in refined cereals^(42,43)^. Data from the national nutrition survey revealed that the a majority of Australians consume less than half of the recommended amount of wholegrain foods and consume an excessive quantity of refined cereal products^(44)^. Similarly in our study, white breads were more frequently consumed than wholegrains. There is a need to raise awareness among parents about increasing their children’s consumption of wholegrain breads/cereals, which offer better health benefits. Notably, in our study, the second primary source of starch intake, excluding the 9-month time point, was cakes/cookies. These foods are typically high in saturated fats and added sugars and have been associated with an increased risk of obesity and chronic diseases and also displace the intake of other healthy core food groups such as vegetables, fruits, wholegrains, dairy and meats^(45,46)^. It is, therefore, important to reduce the intake of starch from discretionary foods in young children as evidence has shown that children who consume these foods at a young age are also likely to consume them later, increasing their risk of developing chronic diseases^(41,47)^.

While tracking of a range of nutrients from early childhood has been reported previously^(22,25)^, our study is the first to assess tracking of total carbohydrates and their subtypes (sugars and starch) from as early as age 9 months to age 5 years. Consistent with existing literature on other nutrients^(22,25)^, we found consistent weak to moderate tracking of total carbohydrate, total sugar and starch intake in early childhood. It is worth highlighting that tracking was observed not only between consecutive time points but also over an extended duration, such as 9 months to 5 years. This may suggest that eating behaviours and taste preferences may develop early, and these in turn may influence dietary intake throughout the life course, underscoring the importance of implementing dietary interventions early in life.

This novel study has several strengths including repeated dietary measurements and assessment of dietary intake through three non-consecutive 24-hour recalls, which yielded high-quality dietary intake data. The generalisability of the study findings may be limited because the study consisted of a high proportion of highly educated mothers (58 %) and first-time mothers. Given added sugar’s potential effect on diet quality and the risk of chronic disease, distinctions between total and added sugars would be informative^(48)^. However, our study did not distinguish between added sugar and total sugar intakes. The primary reason for this was the absence of specific data on added sugars or intrinsic sugars in the food composition tables and databases we utilised (AUSNUT 2007). However, the WHO still supports the importance of assessing ‘total sugar’ since it is the most practical term for describing and measuring sugars, and other terms can pose challenges for analysis and lead to confusion among consumers^(8)^. Recall bias and dietary misreporting are also another possible limitation. However, participants reporting extreme energy intakes were excluded from the current analysis to mitigate the impact of dietary misreporting.

The current study may have the potential to contribute to the refinement of carbohydrate and subtype dietary recommendations in early childhood. This is achieved by offering in-depth insights into early childhood carbohydrate and subtype intake trends. Additionally, identifying the key food sources of carbohydrate and subtype intakes may contribute to dietary monitoring to identify suboptimal sources, providing valuable insights for developing specific dietary guidelines and strategies to improve carbohydrate intake in young children. Our study also suggests that reducing the intake of sugar and starch from discretionary foods and promoting healthier alternatives such as wholegrains in children’s diets is beneficial. However, additional research is required in a nationally representative and diverse population to better understand the changes in carbohydrate intake across early childhood and to identify key food sources. There is also an urgent need to include the assessment of carbohydrate intake in young children, including those aged younger than 2 years, at a national level. A global initiative is necessary to develop more rigorous dietary or nutrient reference values for carbohydrates, sugar and starch. This can be achieved by harmonising the criteria for recommendations and study methodologies related to the intake of various types of carbohydrates, sugars and starch in infants and children as well as collecting similar data across countries. The food regulatory bodies could consider mandating nutritional labelling of food products in Australia to declare the amount and types of specific sugars (added or intrinsic sugars) on packaged foods. Finally, further research will be desirable to distinguish between different types of sugar (added *v*. intrinsic) and various food sources of sugar (e.g. intact fruit *v*. purees) to better understand the sugar intake of children under 5 years of age.

### Conclusion

The present study uses longitudinal data to provide novel evidence on the intake of total carbohydrates and subtypes (sugar, starch) among Australian children aged 9 months to 5 years. Moreover, it offers insight into the contribution of food sources to carbohydrate intakes. These findings may contribute valuable data to inform the refinement of dietary recommendations for total carbohydrate, sugar and starch intakes in young Australian children and the design of early childhood interventions that aim to optimise children’s carbohydrate intake. Further investigation in a nationally representative population is warranted.

## Supporting information

Tesfaye et al. supplementary material Tesfaye et al. supplementary material

## Supplementary material

For supplementary material/s referred to in this article, please visit https://doi.org/10.1017/S0007114524002198
