# Supplementary material for: Carbohydrate intakes, food sources and tracking in Australian young children
Source: Br J Nutr. 2024 Oct 23;132(8):1073–82. doi: 10.1017/S0007114524002198 (PMC11600280; doi:10.1017/S0007114524002198)
Supplement: Tesfaye et al. supplementary material [file S0007114524002198sup001.docx]

| **Supplementary material**  **Table S1.** Categories of total carbohydrate, total sugar, and starch AUSNUT 2007 Food Coding System | |
| --- | --- |
| **Food Group** | **AUSNUT 2007 Food Group Number and Name** |
| **Breast milk** | Separate categories were created for intakes of breast milk and infant/toddler formula |
| **Infant & toddler formula** | 321 Infant Formulae and Human Breast Milk |
| **Milk and milk products** | 191 Dairy Milk (cow, sheep and goat) |
|  | 192 Yoghurt |
|  | 193 Cream |
|  | 194 Cheese |
|  | 195 Frozen Milk Products |
|  | 196 Custards |
|  | 197 Other Dishes Where Milk Or A Milk Product Is The Major Component |
| **Other infant & toddler products** |  |
| **Infant & toddler cereal** | 322 Infant Cereal Products |
| **Infant & toddler food** | 323 Infant Foods |
| **Infant & toddler drink _a_** | 324 Infant Drinks |
| **Breads/Cereals** |  |
| flours, grains | 121 Flours And Other Cereal Grains And Starches |
| regular breads, rolls | 122 Regular Breads, And Bread Rolls (Plain/Unfilled/Untopped Varieties) |
| English muffins; flat, sweet, or savoury breads | 123 English-Style Muffins, Flat Breads, And Savoury and Sweet Breads |
| breakfast cereals, bars | 125 Breakfast Cereals and Bars, Unfortified and Fortified Varieties |
| porridge breakfasts | 126 Breakfast Cereal, Hot Porridge Type |
|  | 135 Mixed Dishes Where Cereal Is The Major Ingredient |
| **pasta** | 124 Pasta And Pasta Products |
| **Cakes/cookies** |  |
| sweet biscuits | 131 Sweet Biscuits |
| savoury biscuits | 132 Savoury Biscuits |
| cakes, buns, muffins | 133 Cakes, Buns, Muffins, Scones, Cake-Type Desserts |
| pastries | 134 Pastries |
|  | 136 Batter-Based Products |
| **Savoury snacks** | 261 Potato Snacks |
|  | 262 Corn snacks |
|  | 263 Extruded Or Reformed Snacks |
|  | 264 Pretzels |
|  | 265 Other Snacks |
| **Sweet snacks** | 273 Dishes And Products Other Than Confectionery Where Sugar |
|  | 281 Chocolate And Chocolate-Based Confectionery |
|  | 282 Cereal-, Fruit-, Nut- And Seed-Bars |
|  | 283 Other Confectionery |
| **Butter/oil/fat spreads** | 141 Butters |
|  | 142 Dairy Blends |
|  | 143 Margarine and Table Spreads |
|  | 144 Vegetable/Nut Oil |
|  | 145 Vegetable based solid and other fats |
|  | 146 Unspecified Fats |
|  | 147 Cream |
| **Beef, veal, lamb** | 181 Muscle Meat |
|  | 187 Mixed Dishes Where Beef, Veal Or Lamb Is The Major Component |
| **Processed meat** | 185 Sausages, Frankfurts And Saveloys |
|  | 186 Processed Meat |
|  | 188 Mixed Dishes Where Pork, Bacon, Ham Is The Major Component |
| **Poultry** | 182 Game And Other Carcase Meats |
|  | 183 Poultry And Feathered Game |
|  | 189 Mixed Dishes Where Poultry Or Game Is The Major Component |
| **Egg and egg dishes** | 171 Eggs |
|  | 172 Dishes Where Egg Is The Major Ingredient |
| **Fish** | 151 Fin Fish (Excluding Commercially Sterile) |
|  | 152 Crustacea And Molluscs (Excluding Commercially Sterile) |
|  | 153 Fish roe |
|  | 154 Packed (Commercially Sterile) Fish And Seafood |
|  | 155 Fish And Seafood Products (Homemade and Takeaway) |
|  | 156 Mixed Dishes With Fish Or Seafood As The Major Component |
| **Potato** | 241 Potato |
| **Legumes** | 251 Mature Legumes And Pulses |
|  | 252 Mature Legume And Pulse Products And Dishes |
|  | 184 Organ Meats And Offal, Products And Dishes |
| **Nuts & seeds** | 221 Seeds And Seed Products |
|  | 222 Nuts And Nut Products |
| **Soup** | 211 Soup (Prepared, Ready to Eat) |
|  | 212 Dry Soup Mix |
| **Fruits** | 161 Pome Fruit |
|  | 162 Berry Fruit |
|  | 163 Citrus Fruit |
|  | 164 Stone Fruit |
|  | 165 Tropical Fruit |
|  | 166 Other Fruit |
|  | 167 Mixtures Of Two Or More Groups Of Fruit |
|  | 168 Dried Fruit, Preserved Fruit |
|  | 169 Mixed Dishes Where Fruit Is The Major Component |
| **Vegetables** | 242 Cabbage, Cauliflower And Similar Brassica Vegetables |
|  | 243 Carrot And Similar Root Vegetables |
|  | 244 Leaf And Stalk Vegetables |
|  | 245 Peas And Beans |
|  | 246 Tomato And Tomato Products |
|  | 247 Other Fruiting Vegetables |
|  | 248 Other Vegetables And Vegetable Combinations |
|  | 249 Dishes Where Vegetable Is The Major Component |
| **Sugar-sweetened beverages** | 113 Fruit And Vegetable Juices, And Drinks |
|  | 114 Cordials |
|  | 115 Soft Drinks, And Flavoured Mineral Waters |
|  | 116 Formulated beverages |
|  | 117 Mineral Waters And Water |
|  | 118 Other Beverage Flavourings and Prepared Beverages |
| **Non-milk drinks** | 111 Tea |
|  | 112 Coffee And Coffee Substitutes |
|  | 202 Soy-Based beverages |
| **Condiments** | 231 Gravies And Savoury Sauces |
|  | 232 Pickles, Chutneys And Relishes |
|  | 233 Salad Dressings |
|  | 234 Stuffings |
|  | 311 Yeast, Yeast, Vegetable And Meat Extracts |
|  | 313 Herbs, Spices, Seasonings And Stock Cubes |
|  | 314 Essences |
|  | 315 Other additives |
| **Sugar-based accompaniments** | 271 Sugar, Honey and Syrups |
|  | 272 Jam and Lemon Spreads, Chocolate Spreads, Sauces |
| **Soy yogurt/ milk substitute** | 201 Dairy Milk Substitutes, Unflavoured |
|  | 203 Cheese Substitute |
|  | 205 Soy-Based Yoghurts |
| **Organ meat** | 184 Liver paste and pate |
| **Dietary product** | 301 Formula Dietary Foods |

^a^ Fruit juices marketed towards infants and toddlers.

Table S2. Comparison of the mean (SD) total carbohydrate, total sugar, and starch (g/d) intake at 9 months, 18 months, 3.5 years, and 5 years by the intervention group in the Melbourne Infant Feeding Activity and Nutrition Trial (InFANT) Program

| Time point | Group | **n^a^** | **Mean (SD) of total CHO** | | **P value^b^** | **Mean (SD) of total sugar** | | **P value** | **Mean (SD) of starch** | | **P value** |
| --- | --- | --- | --- | --- | --- | --- | --- | --- | --- | --- | --- |
| 9 months | Control | 194 | 99.8 | 25.2 | 0.89 | 64.0 | 14.5 | 0.36 | 34.8 | 16.1 | 0.61 |
|  | Intervention | 199 | 99.4 | 26.2 |  | 62.7 | 14.8 |  | 35.7 | 16.2 |  |
| 18 months | Control | 124 | 130.0 | 25.4 | 0.30 | 71.2 | 16.9 | 0.01 | 57.8 | 18.8 | 0.51 |
|  | Intervention | 124 | 126.6 | 28.6 |  | 66.1 | 18.1 |  | 59.3 | 20.3 |  |
| 3.5 years | Control | 125 | 157.6 | 38.2 | 0.67 | 79.4 | 23.4 | 0.49 | 76.6 | 23.4 | 1.0 |
|  | Intervention | 119 | 155.7 | 32.1 |  | 77.4 | 22.2 |  | 76.6 | 20.2 |  |
| 5 years | Control | 118 | 175.5 | 36.8 | 0.56 | 85.6 | 26.4 | 0.13 | 87.8 | 22.4 | 0.39 |
|  | Intervention | 122 | 172.7 | 37.6 |  | 80.6 | 24.8 |  | 90.4 | 24.1 |  |

Table S3. Main total carbohydrate food sources at ages 9 months, 18 months, 3.5 years, and 5 years in Melbourne Infant Feeding Activity and Nutrition Trial (InFANT) Program

|  |  | 9 months (n=393) | | |  | 18 months (n=284) | | | |  | 3.5 years (n=244) | | |  | 5 years (n=240) | | | |
| --- | --- | --- | --- | --- | --- | --- | --- | --- | --- | --- | --- | --- | --- | --- | --- | --- | --- | --- |
|  | % consumers^a^ | % of total energy | % of total CHO | CHO provided (g) | % consumers ^a^ | | % of total energy | % of total CHO | CHO provided (g) | % consumers ^a^ | % of total energy | % of total CHO | CHO provided (g) | % consumers ^a^ | | % of total energy | % of total CHO | CHO provided (g) |
| Food Group |  | Mean (SD) | Mean (SD) | Mean (SD) |  |  | Mean (SD) | Mean (SD) | Mean (SD) |  | Mean (SD) | Mean (SD) | Mean (SD) |  |  | Mean (SD) | Mean (SD) | Mean (SD) |
| Breast milk | 45 | 6.4 (8.5) | 10.5(14.9) | 11.1(13.9) | 10 | | 0.6 (2.5) | 0.8 (3.7) | 1.6 (6.1) | 1 | 0.0 (0.0) | 0.0 (0.0) | 0.0 (0.1) | 0 | | 0 | 0 | 0 |
| Infant/toddler  formula | 70 | 15.0 (12.1) | 24.0 (20.5) | 32.4(26.4) | 13 | | 1.2 (3.8) | 1.5 (4.9) | 3.0 (9.3) | 1 | 0.1 (1.4) | 0.2 (1.9) | 0.4 (4.2) | 1 | | 0.1 (1.3) | 0.1 (1.7) | 0.4 (5.4) |
| Milk and milk products^b^ | 91 | 4.6 (3.9) | 6.9 (5.9) | 9.5 (8.5) | 98 | | 12.9 (6.2) | 17.4 (9.2) | 33.3(16.8) | 98 | 10.9 (5.3) | 13.2 (6.6) | 34.3(18.2) | 98 | | 9.6 (4.9) | 11.1(5.9) | 33.4(18.2) |
| infant drinks^c^ | 4 | 0.1 (0.4) | 0.0 (0.4) | 0.1 (0.7) | 0.3 | | 0.0 (0.3) | 0.0 (0.3) | 0.1(0.7) | 2 | 0.0 (0.2) | 0.0 (0.0) | 0.1 (0.6) | 1 | | 0.0 (0.1) | 0.0 (0.1) | 0.0(0.3) |
| Breads/cereals | 94 | 11.1 (8.8) | 16.1(10.8) | 23.5(21.8) | 100 | | 21.9(10.4) | 28.0(11.5) | 56.6(28.3) | 100 | 25.1(10.7) | 29.3 (1.0) | 77.7(35.7) | 100 | | 26.8 (10.9) | 30.3(10.8) | 91.8(40.8) |
| Pasta | 44 | 2.2 (3.6) | 3.2 (4.9) | 4.7(8.3) | 58 | | 3.8 (4.9) | 4.8 (5.9) | 10.0(13.2) | 57 | 4.5 (5.7) | 5.2 (6.5) | 14.3(18.9) | 55 | | 4.2 (5.4) | 4.6 (5.5) | 14.2(18.1) |
| Infant cereals/products | 76 | 3.4 (3.9) | 5.3 (5.8) | 6.9 (8.5) | 22 | | 0.5 (1.3) | 0.7 (1.7) | 1.3 (3.1) | 1 | 0.0 (0.3) | 0.0 (0.4) | 0.1 (1.0) | 0 | | 0 | 0 | 0 |
| Infant food | 64 | 4.1 (4.4) | 6.2 (6.6) | 8.5 (9.2) | 36 | | 1.6 (2.8) | 2.1 (3.6) | 4.2 (7.2) | 9 | 0.4 (1.2) | 0.5 (1.6) | 1.2 (4.1) | 3 | | 0.1 (0.7) | 0.1(0.9) | 0.3 (2.3) |
| Cakes/cookies | 48 | 1.7 (3.4) | 2.5 (4.7) | 3.7 (8.3) | 88 | | 7.3 (6.9) | 9.2 (8.1) | 19.0(18.8) | 95 | 10.9 (7.7) | 12.7 (8.5) | 34.3(24.6) | 96 | | 11.9 (7.2) | 13.6 (7.9) | 41.6(26.9) |
| Savoury snacks | 2 | 0.0 (0.1) | 0.0 (0.1) | 0.0 (0.2) | 15 | | 3.3 (1.2) | 0.4 (1.5) | 0.8 (3.1) | 76 | 0.8 (2.0) | 1.0 (2.4) | 2.6 (6.2) | 49 | | 1.6 (2.3) | 1.8 (2.7) | 5.5 (8.4) |
| Sweet snacks | 6 | 0.2 (0.9) | 0.3 (1.2) | 0.5 (2.3) | 35 | | 2.3 (4.7) | 2.8 (5.5) | 5.8 (11.7) | 76 | 4.5 (4.9) | 5.2 (5.3) | 13.8(15.3) | 74 | | 4.8 (5.7) | 5.3 (5.7) | 16.4(19.1) |
| Butter/oil/fat spreads | 35 | 0.0 (0.0) | 0.0 (0.1) | 0.0 (0.1) | 78 | | 0.0 (0.1) | 0.0 (0.1) | 0.1 (0.3) | 79 | 0.0 (0.1) | 0.0 (0.1) | 0.1 (0.3) | 85 | | 0.0 (0.3) | 0.1 (0.3) | 6.5 (6.5) |
| Condiments | 45 | 0.2 (0.6) | 0.4 (0.9) | 0.5 (1.4) | 73 | | 0.6 (0.9) | 0.7(1.2) | 1.5 (2.4) | 80 | 0.9 (1.2) | 0.9 (1.2) | 2.6 (3.8) | 85 | | 0.8 (0.9) | 0.9 (1.2) | 2.8 (3.3) |
| Sugar based accompaniments | 8 | 0.1 (0.7) | 0.2 (0.9) | 0.3 (1.5) | 80 | | 0.8 (1.6) | 0.9 (2.0) | 1.9 (4.3) | 51 | 1.6 (2.8) | 1.8 (3.2) | 4.9 (8.8) | 58 | | 2.1 (4.8) | 2.2 (4.1) | 6.9 (13.3) |
| Sugar sweetened beverage | 7 | 0.1 (0.6) | 0.1 (0.8) | 0.2 (0.9) | 23 | | 0.8 (2.1) | 1.1 (2.6) | 1.9 (4.8) | 60 | 3.2 (4.7) | 3.6 (5.2) | 9.7 (14.6) | 67 | | 3.1 (4.0) | 3.4 (4.3) | 10.8(13.8) |
| Beef, veal, lamb | 35 | 0.5 (0.9) | 0.7 (1.6) | 0.9 (2.1) | 49 | | 0.6 (1.2) | 0.8 (1.6) | 1.7 (3.5) | 40 | 0.5 (1.1) | 0.6 (1.4) | 1.6 (3.4) | 39 | | 0.6 (1.2) | 0.7 (1.5) | 2.0 (4.5) |
| Processed meats^d^ | 9 | 0.0 (0.1) | 0.0 (0.2) | 0.1 (0.3) | 30 | | 0.1 (0.3) | 0.2 (0.4) | 0.4 (0.8) | 52 | 0.4 (0.9) | 0.5 (1.0) | 1.3 (2.7) | 49 | | 0.3 (0.6) | 0.3 (0.7) | 0.9 (2.0) |
| Poultry | 24 | 0.5 (1.6) | 0.7 (2.0) | 1.0 (3.9) | 37 | | 0.6 (1.4) | 0.8 (1.8) | 1.5 (3.5) | 41 | 0.8 (1.7) | 0.9 (1.9) | 2.5 (5.8) | 43 | | 1.2 (2.1) | 1.3 (2.4) | 3.9 (7.4) |
| Egg and egg dishes | 10 | 0.1 (0.6) | 0.1 (0.9) | 0.2 (1.6) | 28 | | 0.3 (1.4) | 0.4 (1.6) | 0.9 (4.0) | 21 | 0.2 (1.2) | 2.2 (1.4) | 0.6 (3.6) | 38 | | 0.3 (1.2) | 0.3 (1.5) | 0.9 (4.2) |
| Fish | 10 | 0.12 (0.7) | 0.2 (1.1) | 0.3 (1.5) | 22 | | 0.5 (1.5) | 0.7 (1.8) | 1.4 (3.8) | 52 | 0.5 (1.4) | 0.7 (1.8) | 1.7 (4.6) | 24 | | 0.6 (1.5) | 0.7 (1.7) | 2.3 (5.3) |
| Potato | 42 | 1.3 (2.2) | 1.8 (3.0) | 0.3 (1.5) | 47 | | 1.7 (2.9) | 2.2 (3.8) | 4.5 (7.9) | 67 | 2.9 (3.4) | 3.4 (3.9) | 9.2 (11.1) | 67 | | 2.9 (3.6) | 3.3 (3.9) | 9.8 (11.1) |
| Legumes | 17 | 0.5 (1.5) | 0.6 (2.0) | 0.9 (3.3) | 26 | | 0.9 (2.4) | 1.2 (3.0) | 2.5 (6.4) | 22 | 0.6 (1.5) | 0.6 (1.7) | 1.8 (4.7) | 22 | | 0.7 (1.9) | 0.7 (1.9) | 2.3 (6.2) |
| Nuts & seeds | 2 | 0.0 (0.1) | 0.0 (0.1) | 0.0 (0.1) | 18 | | 0.1 (0.5) | 0.1(0.5) | 0.2 (1.7) | 39 | 0.2 (0.4) | 0.2 (0.4) | 0.6 (1.2) | 35 | | 0.2 (0.5) | 0.3 (0.7) | 0.8 (2.0) |
| Soup | 17 | 0.5 (1.5) | 0.7 (2.1) | 1.1 (3.4) | 22 | | 0.8 (1.9) | 1.0 (2.4) | 2.0 (4.9) | 16 | 0.6 (2.2) | 0.7 (2.3) | 2.1 (8.7) | 20 | | 0.8 (1.9) | 0.9 (2.1) | 2.6 (6.5) |
| Fruit | 95 | 8.6 (6.5) | 12.9 (9.3) | 17.4(13.7) | 99 | | 14.0 (7.0) | 17.9 (8.2) | 36.1(19.1) | 99 | 13.1 (6.7) | 15.3 (7.2) | 40.3(21.8) | 99 | | 12.8 (6.3) | 14.4 (6.7) | 43.2(21.4) |
| Vegetables | 94 | 3.1 (2.9) | 6.1 (5.4) | 8.4 (8.8) | 90 | | 1.4 (1.9) | 3.5 (4.6) | 6.9 (10.9) | 93 | 0.7 (0.8) | 2.2 (2.5) | 6.0 (7.5) | 95 | | 0.8 (0.9) | 2.6 (2.6) | 7.8 (7.8) |

^a^ Percentage of children who consumed food at least once during data collection. ^b^ Includes dairy milk, yoghurt, cheese, frozen milk products and custard. ^c^ Fruit juices marketed towards infants and toddlers ^d^ Includes sausages, ham, bacon, canned meat.

Table S4. Main sugar food sources at ages 9 months, 18 months, 3.5 years, and 5 years in Melbourne Infant Feeding Activity and Nutrition Trial (InFANT) Program

|  |  | 9 months (n=393) | | |  | 18 months (n=284) | | | |  | 3.5 years (n=244) | | |  | 5 years (n=240) | | | |
| --- | --- | --- | --- | --- | --- | --- | --- | --- | --- | --- | --- | --- | --- | --- | --- | --- | --- | --- |
|  | % consumers^a^ | % of total energy | % of total sugar | sugar provided (g) | % consumers | | % of total energy | % of total sugar | sugar provided (g) | % consumers^a^ | % of total energy | % of total sugar | sugar provided (g) | % consumers^a^ | | % of total energy | % of total sugar | sugar provided (g) |
| Food Group |  | Mean (SD) | Mean (SD) | Mean (SD) |  |  | Mean (SD) | Mean (SD) | Mean (SD) |  | Mean (SD) | Mean (SD) | Mean (SD) |  |  | Mean (SD) | Mean (SD) | Mean (SD) |
| Breast milk | 45 | 6.4 (8.5) | 17.9 (23.1) | 11.1(13.9) | 10 | | 0.6 (2.5) | 1.6 (6.3) | 1.6 (6.1) | 1 | 0.0 (0.0) | 0.0 (0.1) | 0.0 (0.1) | 0 | | 0 | 0 | 0 |
| Infant/toddler  formula | 0.3 | 0.0 (0.1) | 0.0 (0.2) | 0.0 (1.1) | 7 | | 0.5 (2.4) | 1.2 (5.7) | 1.2 (5.6) | 1 | 0.1 (1.1) | 0.2 (2.8) | 0.3 (3.4) | 1 | | 0.1 (1.1) | 0.2 (2.5) | 0.3 (4.4) |
| Milk and milk products ^b^ | 91 | 4.4 (3.7) | 18.4 (14.6) | 9.1 (7.9) | 98 | | 12.7 (6.0) | 33.4(15.3) | 32.6(16.2) | 99 | 10.7 (5.2) | 26.7(12.0) | 33.4(17.5) | 98 | | 9.2 (4.6) | 23.3(11.5) | 32.0(17.2) |
| infant drinks ^c^ | 4 | 0.0 (0.3) | 0.2 (1.6) | 0.1 (0.7) | 2 | | 0.0 (0.2) | 0.1 (0.6) | 0.1 (0.7) | 2 | 0.0 (0.2) | 0.1 (0.5) | 0.1 (0.6) | 0.4 | | 0.0 (0.1) | 0.0 (0.3) | 0.0 (0.3) |
| Breads/cereals | 94 | 0.8 (1.1) | 3.7 (4.6) | 1.8 (2.5) | 100 | | 2.5 (2.2) | 6.6 (5.5) | 6.2 (5.2) | 100 | 3.1 (2.4) | 7.7 (5.8) | 9.7 (7.9) | 100 | | 3.3 (2.4) | 8.6 (6.2) | 11.4(8.1) |
| Pasta | 3 | 0.0 (0.0) | 0.0 (0.0) | 0.0 (0.1) | 6 | | 0.0 (0.1) | 0.0 (0.1) | 0.0 (0.1) | 5 | 0.0 (0.0) | 0.0 (0.1) | 0.0 (0.2) | 10 | | 0.0 (0.1) | 0.0 (0.2) | 0.1 (0.3) |
| Infant cereals | 73 | 0.3 (0.6) | 1.6 (4.3) | 0.7 (1.5) | 22 | | 0.1 (0.2) | 0.1 (0.4) | 0.1 (0.4) | 1 | 0.0 (0.0) | 0.0 (0.0) | 0.0 (0.1) | 0 | | 0 | 0 | 0 |
| Infant food | 63 | 2.4 (2.9) | 10.8 (13.5) | 4.9 (6.2) | 35 | | 0.9 (1.8) | 2.2 (4.6) | 2.2 (4.7) | 9 | 0.3 (1.1) | 0.8 (3.2) | 0.9 (3.6) | 3 | | 0.1 (0.5) | 0.2 (1.6) | 0.2 (1.5) |
| Cakes/cookies | 48 | 0.3 (0.9) | 1.7 (5.3) | 0.9 (3.5) | 88 | | 1.1 (1.4) | 5.5 (7.2) | 5.7 (8.3) | 95 | 1.3 (1.3) | 8.5 (7.6) | 11.1(10.9) | 96 | | 1.3 (1.1) | 9.6 (8.4) | 13.6(12.7) |
| Savoury snacks | 2 | 0.0 (0.0) | 0.0 (0.0) | 0.0 (0.0) | 15 | | 0.0 (0.1) | 0.0 (0.2) | 0.0 (0.2) | 25 | 0.0 (0.1) | 0.1 (0.4) | 0.1 (0.5) | 49 | | 0.1 (0.1) | 0.2 (0.3) | 0.2 (0.4) |
| Sweet snacks | 6 | 0.2 (0.9) | 0.7 (4.0) | 0.4 (1.9) | 35 | | 1.5 (3.2) | 3.6 (7.5) | 3.6 (7.6) | 75 | 3.3 (3.7) | 3.8 (3.9) | 9.9 (11.0) | 73 | | 3.5 (4.3) | 8.2 (8.8) | 12 (14.4) |
| Butter/oil/fat spreads | 35 | 0.0(0.0) | 0.0 (0.1) | 0.0 (0.1) | 78 | | 0.0(0.1) | 0.1 (0.2) | 0.1 (0.3) | 79 | 0.0 (0.1) | 0.1 (0.3) | 0.1 (0.3) | 85 | | 0.0 (0.3) | 0.1 (0.5) | 0.2 (1.1) |
| Condiments | 43 | 0.1(0.4) | 0.5 (1.6) | 0.3 (0.9) | 72 | | 0.3 (0.8) | 0.8 (2.0) | 0.8 (1.9) | 78 | 0.6 (0.9) | 1.4 (2.2) | 1.9 (3.3) | 83 | | 0.6 (0.9) | 1.5 (2.3) | 1.9 (2.8) |
| Sugar based accompaniments | 8 | 0.1 (0.7) | 0.5 (2.6) | 0.3 (1.5) | 36 | | 0.8 (1.7) | 1.8 (3.7) | 1. 9 (4.3) | 52 | 1.6 (2.8) | 3.7 (5.9) | 4.9 (8.8) | 58 | | 2.1 (4.8) | 4.4 (6.9) | 6.9 (13.3) |
| Sugar sweetened beverage | 7 | 0.1 (0.6) | 0.3 (1.6) | 0.2 (0.9) | 23 | | 0.8 (2.0) | 2.0 (4.9) | 1.9 (4.8) | 59 | 3.1 (4.7) | 7.1 (9.7) | 9.6 (14.5) | 67 | | 3.0 (4.0) | 6.9 (8.3) | 10.5(13.6) |
| Soy yogurt substitute | 5 | 0.1 (0.7) | 0.5 (3.5) | 0.3 (1.6) | 6 | | 0.2 (1.0) | 0.6 (3.7) | 0.5(2.6) | 9 | 0.2 (0.7) | 0.4 (1.6) | 0.5 (2.1) | 8 | | 0.2 (0.9) | 0.4 (2.2) | 0.5(2.7) |
|  |  |  |  |  |  | |  |  |  |  |  |  |  |  | |  |  |  |
| Beef, veal, lamb | 36 | 0.3 (0.5) | 1.2 (2.3) | 0.6 (1.1) | 47 | | 0.3 (0.8) | 0.9 (2.0) | 0.9 (2.0) | 39 | 0.3 (0.5) | 0.7 (1.7) | 0.8 (1.6) | 38 | | 0.3 (0.5) | 0.8 (1.4) | 0.9(1.8) |
| Processed meats ^d^ | 6 | 0.0 (0.1) | 0.0 (0.1) | 0.0 (0.0) | 24 | | 0.0 (0.0) | 0.0 (0.1) | 0.0 (0.1) | 45 | 0.1 (0.2) | 0.1 (0.4) | 0.2 (0.5) | 45 | | 0.1 (0.4) | 0.2 (1.0) | 0.3(1.3) |
| Poultry | 24 | 0.2 (0.7) | 0.9 (2.8) | 0.5 (1.8) | 37 | | 0.2 (0.4) | 0.4 (1.1) | 0.4 (1.1) | 41 | 0.1 (0.3) | 0.3 (1.1) | 0.4 (1.1) | 42 | | 0.3 (0.9) | 0.7 (2.1) | 0.9 (2.9) |
| Egg and egg dishes | 10 | 0.0 (0.1) | 0.0 (0.4) | 0.0 (0.2) | 28 | | 0.1 (0.2) | 0.2 (0.5) | 0.2 (0.5) | 36 | 0.0 (0.1) | 0.1 (0.4) | 0.1 (0.4) | 38 | | 0.1 (0.2) | 0.2 (2.5) | 0.2 (0.5) |
| Fish | 8 | 0.0 (0.2) | 0.1 (1.2) | 0.1 (0.5) | 19 | | 0.1 (0.2) | 0.2 (0.7) | 0.2 (0.7) | 18 | 0.1 (0.2) | 0.2 (0.9) | 0.2 (0.6) | 20 | | 0.1 (0.3) | 0.2 (2.7) | 0.3 (0.9) |
| Potato | 42 | 0.1 (0.2) | 0.5 (1.1) | 0.2 (0.5) | 47 | | 0.1 (0.2) | 0.3 (0.5) | 0.3 (0.5) | 67 | 0.2 (0.2) | 0.4 (0.5) | 0.5 (0.6) | 67 | | 0.2 (0.2) | 0.4 (0.6) | 0.5 (0.6) |
| legumes | 17 | 5.1 (20.2) | 0.6 (2.2) | 0.3 (1.2) | 25 | | 0.3 (0.8) | 0.9 (2.3) | 0.8 (2.2) | 20 | 0.2 (0.5) | 0.4 (1.3) | 0.5 (1.6) | 21 | | 0.2 (0.7) | 0.5 (1.7) | 0.7 (2.1) |
| Nuts & seeds | 2 | 0.0 (0.0) | 0.0 (0.3) | 0.0 (0.1) | 18 | | 0.0 (0.1) | 0.1 (0.3) | 0.1 (0.3) | 39 | 0.1 (0.2) | 0.2 (0.5) | 0.3 (0.6) | 35 | | 0.1 (0.2) | 0.3 (0.6) | 0.7 (2.1) |
| soup | 16 | 0.2 (0.5) | 0.7 (2.2) | 0.3 (1.1) | 21 | | 0.2 (0.8) | 0.6 (1.9) | 0.6 (1.9) | 16 | 0.2 (0.9) | 0.6 (2.4) | 0.7 (2.9) | 18 | | 0.2 (0.7) | 0.5 (1.7) | 0.7 (2.3) |
| Fruit | 95 | 7.6 (5.7) | 29.6 (18.2) | 15.3(12.2) | 99 | | 12.8 (6.5) | 33.1(14.2) | 32.9(17.7) | 99 | 11.9 (6.1) | 29.2(13.4) | 36.8(20.0) | 99 | | 11.6 (5.8) | 28.5(12.3) | 39.4(19.9) |
| Vegetables | 94 | 2.2 (1.9) | 9.5 (8.5) | 0.2 (0.9) | 89 | | 1.2 (1.3) | 3.4 (3.9) | 3.1 (3.3) | 93 | 1.1 (1.1) | 2.8 (2.8) | 3.5 (7.5) | 95 | | 1.5 (1.2) | 3.8 (3.1) | 4.9 (4.1) |

^a^ Percentage of children who consumed food at least once during data collection. ^b^ Includes dairy milk, yoghurt, cheese, frozen milk products and custard. ^c^ Fruit juices marketed towards infants and toddlers ^d^ Includes sausages, ham, bacon, canned meat

Table S5. Main starch food sources at age 9 months, 18 months, 3.5 years, and 5 years in Melbourne Infant Feeding Activity and Nutrition Trial (InFANT) Program

|  |  | 9 months (n=393) | | |  | 18 months (n=284) | | | |  | 3.5 years (n=244) | | |  | 5 years (n=240) | | | |
| --- | --- | --- | --- | --- | --- | --- | --- | --- | --- | --- | --- | --- | --- | --- | --- | --- | --- | --- |
|  | % consumers^a^ | % of total energy | % of starch | starch provided (g) | % consumers | | % of total energy | % of starch | starch provided (g) | % consumers | % of total energy | % of total starch | starch provided (g) | % consumers | | % of total energy | % of starch | Starch provided (g) |
| Food Group |  | Mean (SD) | Mean (SD) | Mean (SD) |  |  | Mean (SD) | Mean (SD) | Mean (SD) |  | Mean (SD) | Mean (SD) | Mean (SD) |  |  | Mean (SD) | Mean (SD) | Mean (SD) |
| Breast milk | 45 | 0.0 (0.0) | 0.0 (0.0) | 0.0(0.0) | 0 | | 0.0 (0.0) | 0.0 (0.0) | 0.0 (0.0) | 0 | 0.0 (0.0) | 0.0 (0.0) | 0.0 (0.0) | 0 | | 0 | 0 | 0 |
| Infant/toddler  formula | 0.3 | 0.0 (0.0) | 0.0 (0.1) | 0.0 (0.0) | 7 | | 0.1 (0.5) | 0.3 (1.5) | 0.3 (1.2) | 1 | 0.0 (0.2) | 0.1 (0.8) | 0.1 (0.8) | 1 | | 0.0 (0.2) | 0.0 (0.3) | 0.1 (0.9) |
| Milk and milk products ^b^ | 29 | 0.2 (0.5) | 1.1 (3.6) | 0.4 (1.1) | 43 | | 0.3 (0.5) | 0.8 (1.6) | 0.7 (1.3) | 67 | 1.3 (2.7) | 2.9 (5.7) | 4.4 (9.1) | 64 | | 0.0 (0.0) | 0.4 (0.6) | 1.2 (2.1) |
| Dietary product | 0 | 0.0 (0.0) | 0.0 (0.0) | 0.0 (0.0) | 1 | | 0.0 (0.1) | 0.0 (0.2) | 0.0 (0.3) | 2 | 0.1 (0.9) | 0.3 (2.0) | 0.4 (3.1) |  | | 0.0 (0.0) | 0.0 (0.5) | 0.2 (2.2) |
| infant drinks ^c^ | 3 | 0.0 (0.0) | 0.0 (0.1) | 0.0 (0.0) | 2 | | 0.0 (0.0) | 0.0 (0.0) | 0.0 (0.0) | 0 | 0.0 (0.0) | 0.0 (0.0) | 0.0 (0.0) | 0.0 | | 0.0 (0.0) | 0.0 (0.0) | 0.0 (0.0) |
| Breads/cereals | 94 | 8.9 (6.7) | 44.6 (21.9) | 18.7(16.2) | 100 | | 19.3 (9.3) | 52.5(17.0) | 49.9(25.4) | 100 | 22.0 (9.3) | 49.1(16.4) | 67.8(30.4) | 98 | | 23.4 (9.7) | 26.4(9.5) | 80.1(36.2) |
| Pasta | 44 | 2.2 (3.6) | 9.9 (15.0) | 4.7 (8.2) | 56 | | 3.5 (4.9) | 9.3 (11.4) | 9.4 (12.9) | 57 | 4.5 (5.7) | 9.6 (11.5) | 14.3(18.8) | 55 | | 4.2 (5.4) | 8.4 (9.9) | 14.1(18.0) |
| Infant cereals | 44 | 1.2 (2.3) | 7.7 (12.8) | 2.5 (5.2) | 7 | | 0.1 (0.9) | 0.5 (2.8) | 0.3 (1.8) | 1 | 0.0 (0.3) | 0.0 (0.6) | 0.1 (1.0) | 0 | | 0 | 0 | 0 |
| Infant food | 41 | 0.4 (0.9) | 2.8 (8.8) | 0.8 (1.8) | 14 | | 0.1 (0.5) | 0.3 (1.7) | 0.2 (1.3) | 9 | 0.1 (0.4) | 0.2 (1.1) | 0.2 (1.2) | 3 | | 0.0 (0.3) | 0.0 (0.3) | 0.1 (1.0) |
| Cakes/cookies | 48 | 1.0 (1.6) | 6.8 (11.1) | 2.8 (5.5) | 88 | | 2.6 (2.3) | 13.8(11.3) | 13.2(12.3) | 95 | 2.9 (2.1) | 16.6(10.8) | 23.2(16.2) | 96 | | 2.7 (1.7) | 17.7(10.1) | 28.0(17.6) |
| Savoury snacks | 2 | 0.0 (0.1) | 0.1 (0.7) | 0.0 (0.2) | 15 | | 0.3 (1.2) | 0.8 (2.9) | 0.8 (3.0) | 25 | 0.8 (1.9) | 1.8 (4.4) | 2.4 (5.7) | 49 | | 1.5 (2.2) | 3.3 (5.0) | 5.3 (8.1) |
| Sweet snacks | 3 | 0.0 (0.2) | 0.1 (0.7) | 0.1 (0.6) | 31 | | 0.7 (1.6) | 2.1 (4.6) | 1.9 (4.3) | 71 | 0.9 (1.5) | 2.2 (3.6) | 3.0 (4.8) | 70 | | 1.0 (1.5) | 2.2 (3.5) | 3.3 (5.1) |
| Butter/oil/fat spreads | 0 | 0.0 (0.0) | 0.0 (0.0) | 0.0 (0.0) | 0 | | 0.0 (0.0) | 0.0 (0.0) | 0.0 (0.0) | 0 | 0.0 (0.0) | 0.0 (0.0) | 0.0 (0.0) | 0 | | 0.0 (0.0) | 0.0 (0.0) | 0.0 (0.0) |
| Condiments | 45 | 0.1 (0.3) | 0.7 (1.6) | 0.3 (0.6) | 70 | | 0.3 (0.3) | 0.7 (0.9) | 0.7 (0.8) | 80 | 0.3 (0.3) | 0.6 (0.7) | 0.8 (0.9) | 83 | | 0.3 (0.3) | 0.6 (0.6) | 0.9 (0.9) |
| Sugar based accomp | 5 | 0.0 (0.0) | 0.0 (0.0) | 0.0 (0.0) | 22 | | 0.0 (0.0) | 0.0 (0.0) | 0.0 (0.0) | 24 | 0.0 (0.1) | 0.0 (0.2) | 0.0 (0.1) | 30 | | 0.0(0.0) | 0.0 (0.0) | 0.0 (0.1) |
| Sugar sweet beverage | 2 | 0.0 (0.0) | 0.0 (0.0) | 0.0 (0.0) | 5 | | 0.0 (0.1) | 0.0 (0.3) | 0.0 (0.2) | 19 | 0.0 (0.1) | 0.1 (0.3) | 0.1 (0.3) | 29 | | 0.0 (0.1) | 0.1 (0.3) | 0.2 (0.5) |
| Soy yogurt substitute | 5 | 0.1 (0.5) | 0.4 (2.5) | 0.2 (1.3) | 6 | | 0.3 (1.6) | 0.7 (3.4) | 0.8 (4.3) | 9 | 0.3 (1.1) | 0.7 (2.6) | 0.8 (3.1) | 8 | | 0.3 (1.0) | 0.5 (2.2) | 0.9 (3.3) |
|  |  |  |  |  |  | |  |  |  |  |  |  |  |  | |  |  |  |
| Beef, veal, lamb | 35 | 0.2 (0.6) | 0.7 (1.6) | 0.4 (1.2) | 49 | | 0.2 (0.6) | 0.7 (2.0) | 0.7 (1.9) | 38 | 0.2 (0.7) | 0.6 (1.8) | 0.7 (2.1) | 38 | | 0.3 (1.0) | 0.7 (2.1) | 1.0 (3.6) |
| Processed meats ^d^ | 6 | 0.0 (0.1) | 0.1 (0.7) | 0.0 (0.3) | 25 | | 0.1 (0.3) | 0.4 (0.9) | 0.3 (0.8) | 42 | 0.3 (0.8) | 0.8 (1.7) | 1.1(2.1) | 43 | | 0.2 (0.4) | 0.5 (0.8) | 0.7(1.3) |
| Poultry | 20 | 0.2 (1.1) | 1.2 (4.2) | 0.6 (2.7) | 31 | | 0.4 (1.2) | 1.2 (3.4) | 1.1 (3.0) | 35 | 0.7 (1.5) | 1.5 (3.2) | 2.1 (5.2) | 38 | | 0.9 (1.7) | 2.0 (0.7) | 3.1 (5.9) |
| Egg and egg dishes | 2 | 0.1(1.6) | 0.3 (2.6) | 0.2 (1.4) | 6 | | 0.3 (1.2) | 0.6 (2.9) | 0.7 (3.5) | 3 | 0.1 (1.0) | 0.3 (2.2) | 0.5 (3.1) | 5 | | 0.2 (1.0) | 0.5 (2.5) | 0.7 (3.6) |
| Fish | 6 | 0.1(0.5) | 0.5 (3.1) | 0.2 (1.1) | 19 | | 0.5 (1.4) | 1.2 (3.4) | 1.2 (3.5) | 18 | 0.5 (1.3) | 1.1 (3.1) | 1.5 (4.2) | 22 | | 0.6 (1.4) | 1.1 (2.6) | 2.0 (4.8) |
| Potato | 42 | 1.2 (2.0) | 6.0 (10.0) | 2.4 (4.4) | 48 | | 1.6 (2.8) | 4.5 (7.4) | 4.2 (7.5) | 67 | 2.8 (3.3) | 6.2 (7.3) | 8.7 (10.8) | 67 | | 2.8 (3.5) | 5.9 (6.9) | 9.2 (10.8) |
| legumes | 17 | 0.3 (0.9) | 1.6 (5.1) | 0.6 (2.1) | 26 | | 0.6 (1.5) | 1.6 (4.1) | 1.6 (4.1) | 22 | 0.4 (1.0) | 0.9 (2.3) | 1.2 (3.0) | 22 | | 0.4 (1.3) | 0.9 (2.5) | 1.5 (4.0) |
| Nuts & seeds | 2 | 0.0 (0.0) | 0.0 (0.1) | 0.0 (0.0) | 6 | | 0.0 (0.4) | 0.1 (0.9) | 0.1 (1.5) | 36 | 0.1 (0.2) | 0.2 (0.5) | 0.3 (0.7) | 32 | | 0.1 (0.3) | 0.3 (0.8) | 0.4 (1.3) |
| soup | 17 | 0.3 (1.1) | 1.9 (5.9) | 0.3(2.6) | 22 | | 0.5 (1.4) | 1.5 (3.8) | 1.3 (3.5) | 16 | 0.4 (1.6) | 0.8 (2.6) | 1.3 (6.8) | 20 | | 0.6 (1.6) | 1.1 (3.1) | 1.9 (5.2) |
| Fruit | 86 | 0.5 (0.5) | 3.4 (4.7) | 1.0(1.1) | 97 | | 0.8 (0.6) | 2.4 (1.8) | 2.1 (1.4) | 94 | 0.7 (0.6) | 1.7 (1.5) | 2.2 (1.7) | 94 | | 0.7 (0.5) | 1.6 (1.2) | 2.4(1.7) |
| Vegetables | 87 | 1.6 (2.1) | 9.6 (11.3) | 3.4 (4.8) | 79 | | 1.5 (3.3) | 3.8 ( 6.6) | 3.7 (9.2) | 79 | 0.8 (1.4) | 1.8 (3.3) | 2.4 (4.9) | 78 | | 0.8 (1.4) | 1.8 (2.9) | 2.6 (4.4) |

^a^ Percentage of children who consumed food at least once during data collection. ^b^ Includes dairy milk, yoghurt, cheese, frozen milk products and custard. ^c^ Fruit juices marketed towards infants and toddlers ^d^ Includes sausages, ham, bacon, canned meat

Table S6. Tracking of total carbohydrate, sugar, and starch at ages 9 months, 18 months, 3.5 years, and 5 years in Melbourne Infant Feeding Activity and Nutrition Trial (InFANT) Program

|  | **9 months** | **18 months** | **3.5 years** | **5 years** |
| --- | --- | --- | --- | --- |
|  | **Tracking (p- value) ^a^** | **Tracking (p-value)** | **Tracking (p-value)** | **Tracking (p-value)** |
|  | CHO Sugar Starch | CHO Sugar Starch | CHO Sugar Starch | CHO Sugar Starch |
| **18 months** |  |  |  |  |
| CHO | 0.27 (P<0.01) |  |  |  |
| Sugar  Starch | 0.10 (0.23)  0.12 (0.15) |  |  |  |
| **3.5 years** |  |  |  |  |
| CHO | 0.21(0.01) | 0.26 (P<0.01) |  |  |
| Sugar  Starch | 0.09 (0.30)  0.16 (0.06) | 0.26 (P<0.01)  0.35 (P<0.0001) |  |  |
| **5 years** |  |  |  |  |
| CHO | 0.01 (0.85) | 0.12 (0.17) | 0.42 (P<0.0001) |  |
| Sugar  Starch | 0.16 (0.05)  0.02 (0.86) | 0.27 (P<0.001)  0.19 (0.02) | 0.42 (P<0.0001)  0.43(P<0.0001) |  |

CHO, Total carbohydrates, ^a^Pearson correlation of linear regression predicted residuals of carbohydrates, total sugar and starch at each timeline, n=141 (children with data at all four time points)

Figure S1. Flow chart showing the number of participants included in the analysis of Carbohydrate Intakes, Food Sources and Tracking in Australian young children
